# Impact of multimodal analgesia nursing on postoperative pain and recovery outcomes after hepatectomy

**DOI:** 10.1097/MD.0000000000047526

**Published:** 2026-02-13

**Authors:** Qi Jiang, Ye’ang Qin

**Affiliations:** a Department of Hepatobiliary Surgery, The First People’s Hospital of Changde City, Changde City, Hunan Province, China.

**Keywords:** complications, hepatectomy, multimodal analgesia, nursing intervention, pain management, postoperative recovery

## Abstract

This study aims to investigate the impact of multimodal analgesia (MMA) nursing on postoperative pain management and recovery outcomes in patients undergoing hepatectomy. A retrospective cohort study was conducted involving 120 patients who underwent elective hepatectomy between January 2023 and January 2024. Patients were divided into a conventional analgesia nursing group (n = 60) and a MMA nursing group (n = 60). Postoperative pain intensity (Visual Analogue Scale), analgesic consumption, recovery-related indicators, complication rates, and patient-reported outcomes were compared between groups. Baseline characteristics were comparable between groups (*P* > .05). Visual Analogue Scale scores at 6, 12, 24, and 48 hours postoperatively were significantly lower in the MMA nursing group (*P* < .001). The MMA group also demonstrated reduced analgesic consumption, earlier ambulation and bowel-function recovery, shorter hospital stays, and a lower incidence of postoperative complications (*P* < .05). Patient satisfaction, compliance, and pain-management knowledge were significantly improved. Multimodal analgesia nursing effectively improves postoperative pain control, accelerates recovery, reduces complications, and enhances patient-reported outcomes after hepatectomy, supporting its clinical value within Enhanced Recovery After Surgery-based perioperative care.

## 1. Introduction

Hepatectomy remains a major surgical procedure for the treatment of both primary and metastatic liver tumors; however, it is often associated with significant postoperative pain and a complex recovery process. Severe incisional and traction pain can impair deep breathing, effective coughing, and early ambulation, consequently delaying bowel-function recovery, increasing the risk of complications, prolonging hospitalization, and reducing overall patient satisfaction.^[1–3]^ Conventional postoperative analgesia primarily relies on opioid administration, which, although effective for pain control, frequently induces adverse effects such as nausea, vomiting, respiratory depression, sedation, and reduced bowel motility, all of which hinder postoperative recovery.^[4]^ To optimize perioperative pain management, the concept of Enhanced Recovery After Surgery (ERAS) has been widely applied in hepatobiliary surgery. The ERAS protocol emphasizes minimizing opioid use and implementing multimodal analgesia (MMA) strategies to promote early rehabilitation.^[5]^

MMA integrates multiple analgesic modalities that act through different mechanisms, including acetaminophen, nonsteroidal anti-inflammatory drugs (NSAIDs), local anesthetics, α_2_-adrenergic receptor agonists, and N-methyl-D-aspartate receptor antagonists, along with regional nerve blocks, psychological interventions, and comfort-oriented nursing care. These measures act synergistically on various pain transmission pathways, thereby enhancing analgesic efficacy while reducing opioid requirements and related adverse effects.^[6]^

In 2021, the Procedure-Specific Postoperative Pain Management expert consensus on open hepatectomy recommended baseline use of acetaminophen and NSAIDs in combination with thoracic epidural analgesia or transversus abdominis plane block, with systemic opioids reserved only for rescue analgesia. Subsequently, the 2022 ERAS guidelines for liver surgery provided high-level evidence and strong recommendations supporting MMA throughout the perioperative period and incorporated it into standardized recovery pathways.

Furthermore, Hardman et al^[4]^ demonstrated in living liver donors that implementation of an MMA regimen significantly reduced postoperative opioid consumption and pain scores. Similarly, Kianian et al^[3]^ reviewed that MMA not only effectively alleviates postoperative pain but also improves the quality of functional recovery. Recent evidence-based reviews have confirmed that acetaminophen and NSAIDs are safe and effective after hepatectomy and constitute essential components of the MMA strategy.

Despite these advances, systematic studies focusing on MMA nursing pathways remain limited. Most existing studies have concentrated on anesthetic or pharmacologic aspects, with insufficient evaluation of the combined effects of nursing interventions-such as pain education, psychological support, and early ambulation guidance-within MMA-based frameworks. Therefore, the present study retrospectively analyzed patients undergoing elective hepatectomy to compare MMA nursing with conventional analgesia nursing in terms of postoperative pain, recovery rate, complication incidence, and patient-reported outcomes. These findings aim to provide clinical evidence for optimizing perioperative pain management and nursing pathways in hepatic surgery.

## 2. Materials and methods

### 2.1. Study design and participants

This study was approved by the Ethics Committee of The First People’s Hospital of Changde City(Approval No. 2023-HB-017). This single-center retrospective cohort study evaluated the effects of MMA nursing on postoperative pain and recovery after hepatectomy. The study complied with the Declaration of Helsinki; written informed consent was waived by the institutional ethics committee due to the retrospective design.

A total of 120 patients who underwent elective hepatectomy in our hepatobiliary surgery department from January 2023 to January 2024 were included. Based on postoperative analgesia nursing strategies, patients were assigned to a control group (n = 60; conventional analgesia nursing) or an intervention group (n = 60; MMA nursing). To minimize selection bias, all consecutive patients who met the predefined inclusion and exclusion criteria during the study period were enrolled. Information bias was reduced by using standardized electronic medical records, unified nursing documentation, and validated assessment tools (Visual Analogue Scale [VAS]). Data extraction was independently performed by 2 trained investigators, and discrepancies were resolved through discussion. Patients were assigned to groups based on the postoperative analgesia nursing strategy implemented during different time periods. Conventional analgesia nursing was applied in the earlier phase, whereas MMA nursing was introduced later following updates to the clinical nursing pathway. Therefore, group allocation was nonrandom and based on a temporal sequence.

### 2.2. Inclusion and exclusion criteria

#### 2.2.1. Inclusion criteria

1. Age 18 to 75 years.
2. Indication for hepatectomy confirmed by preoperative imaging.
3. American Society of Anesthesiologists (ASA) physical status class I to III.
4. General anesthesia.
5. Complete clinical data.

#### 2.2.2. Exclusion criteria

1. Severe preexisting cardiac, pulmonary, or renal dysfunction.
2. Psychiatric or neurological disorders or communication barriers.
3. Conversion to emergency surgery or concurrent major procedures.
4. Reoperation or intensive care unit stay > 48 hour, postoperatively.
5. Incomplete data or loss to follow-up.

### 2.3. Nursing interventions

#### 2.3.1. Control group (conventional analgesia nursing)

Routine postoperative patient-controlled intravenous analgesia was administered. The solution contained sufentanil 2 μg/mL and flurbiprofen axetil 50 mg/50 mL; background 2 mL/h, bolus 2 mL, lockout 15 minutes. Standard pain assessment and basic nursing education were provided.

#### 2.3.2. Intervention group (MMA nursing)

On the basis of routine care, comprehensive MMA nursing included:

1. Pharmacologic optimization: intraoperative combination of acetaminophen, NSAIDs (flurbiprofen axetil), and local anesthetic (0.375% ropivacaine) via transversus abdominis plane block; within 24 hours postoperatively, combined intravenous and oral analgesics were used to minimize opioid requirements.
2. Non-pharmacologic analgesia: within 6 hours postoperatively, cold compresses, position adjustment, relaxation guidance, and music therapy; at 12 hours, instruction in diaphragmatic breathing.
3. Pain assessment and dynamic management: pain was assessed every 6 hours using the VAS, with analgesic and non-pharmacologic measures adjusted according to scores.
4. Early rehabilitation nursing: with adequate analgesia, in-bed turning and lower-limb pump exercises were guided, ambulation was encouraged within 24 hours, and early bowel-function care was implemented.
5. Health education and psychological support: preoperative education on pain management and continuous postoperative psychological support to enhance adherence and self-management.

### 2.4. Observation indicators

1. General information: sex, age, body mass index, ASA class, surgical type, operation time, and intraoperative blood loss.
2. Pain score: VAS at 6, 12, 24, and 48 hours postoperatively.
3. Recovery indicators: total analgesic consumption within 48 hours, time to 1st ambulation, time to bowel-function recovery, and length of hospital stay.
4. Complications: incidence of nausea/vomiting, respiratory depression, and wound infection within 48 hours after surgery.
5. Patient-reported outcomes: postoperative satisfaction (0–10), nursing compliance score, and rate of pain-management knowledge acquisition.

### 2.5. Statistical analysis

Data were analyzed using Statistical Package for the Social Sciences (SPSS version 26.0) (IBM, Armonk). Continuous variables were tested for normality and expressed as mean ± SD (x ± s); between-group comparisons used independent-samples *t* tests. Categorical variables were presented as n (%) and compared using the *χ*^2^ test or Fisher exact test. Two-tailed *P* < .05 was considered statistically significant.

## 3. Results

### 3.1. Baseline characteristics

There were no significant between-group differences in age, sex, body mass index, ASA classification, surgical type, operation time, or intraoperative blood loss (*P* > .05), indicating good comparability (Table 1).

**Table 1:** Comparison of baseline characteristics.

Variable	Control group (n = 60)	Intervention group (n = 60)	*t*/χ^2^	*P* value
Age (yr)	56.30 ± 9.10	55.80 ± 8.70	0.272	.786
Sex (M/F)	35/25	33/27	0.066	.797
BMI (kg/m^2^)	23.80 ± 2.60	23.50 ± 2.80	0.471	.639
ASA classification (I/II/III)	12/38/10	14/36/10	0.147	.930
Surgical type (left/right/central)	20/32/8	19/33/8	0.050	.974
Operation time (min)	190.00 ± 35.00	185.00 ± 37.00	0.701	.485
Intraoperative blood loss (mL)	350.00 ± 100.00	340.00 ± 110.00	0.471	.639

BMI = body mass index.

### 3.2. Postoperative pain scores (VAS)

**Figure 1.**
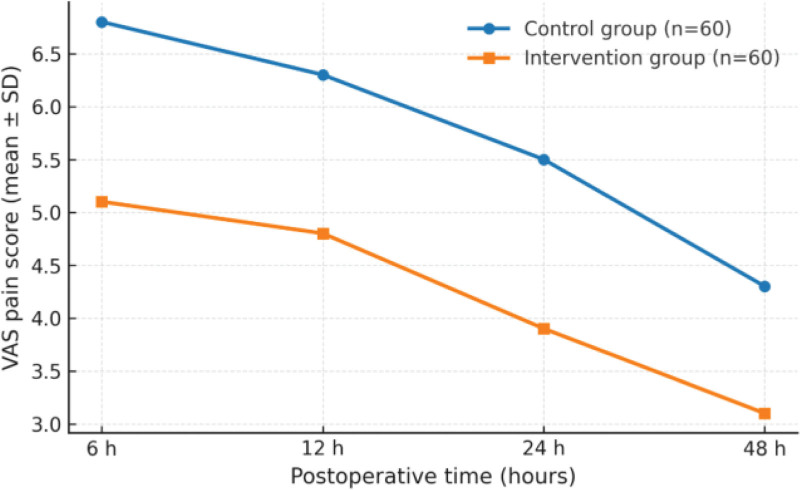
Postoperative VAS trends between groups. VAS = Visual Analogue Scale.

At 6, 12, 24, and 48 hours after surgery, VAS scores were consistently lower in the intervention group than in the control group (all *P* < .001), indicating that MMA nursing attenuated early postoperative pain responses (Table 2; Fig. 1).

**Table 2:** Postoperative pain scores (VAS) at different time points.

Time point	Control group	Intervention group	*t* value	*P* value
6 h	6.80 ± 1.00	5.10 ± 0.90	9.235	<.001
12 h	6.30 ± 0.80	4.80 ± 1.00	8.365	<.001
24 h	5.50 ± 0.90	3.90 ± 0.70	10.02	<.001
48 h	4.30 ± 0.80	3.10 ± 0.60	9.762	<.001

VAS = Visual Analogue Scale.

### 3.3. Postoperative recovery indicators

**Figure 2.**
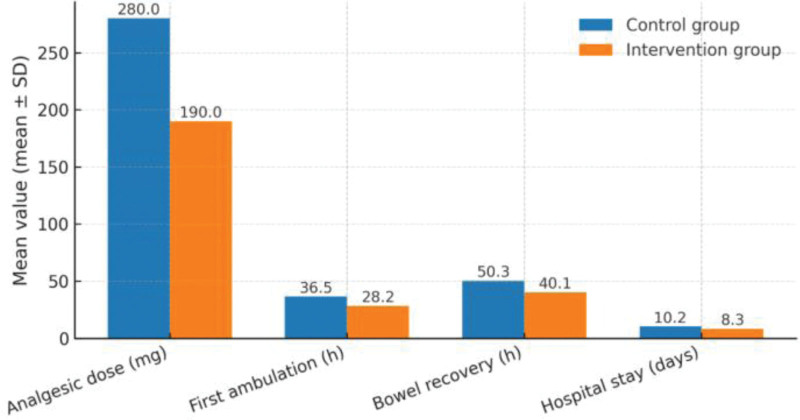
Comparison of postoperative recovery indicators.

Compared with the control group, the intervention group had a lower total analgesic consumption within 48 hours and shorter times to 1st ambulation, bowel-function recovery, and hospital stay (all *P* < .001) (Table 3; Fig. 2).

**Table 3:** Postoperative recovery indicators.

Indicator	Control group	Intervention group	*t* value	*P* value
Total analgesic dose (48 h, mg)	280.00 ± 65.00	190.00 ± 50.00	8.202	<.001
Time to 1st ambulation (h)	36.50 ± 8.30	28.20 ± 7.90	5.812	<.001
Time to bowel-function recovery (h)	50.30 ± 10.40	40.10 ± 9.50	5.324	<.001
Length of hospital stay (d)	10.20 ± 2.10	8.30 ± 1.80	5.139	<.001

### 3.4. Postoperative complications

**Figure 3.**
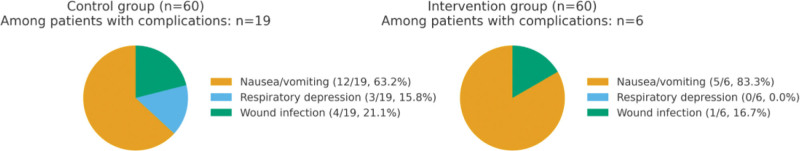
Distribution of postoperative complications (excluding patients without complications).

The overall complication rate was 31.67% (19/60) in the control group and 10.00% (6/60) in the intervention group (χ^2^ = 8.557, *P* = .003). Rates of nausea/vomiting and wound infection were lower in the intervention group, and no respiratory depression occurred in that group (Table 4; Fig. 3).

**Table 4:** Postoperative complication incidence.

Complication	Control group	Intervention group	χ^2^ value	*P* value
Nausea/vomiting	12 (20.00%)	5 (8.33%)	3.981	.046
Respiratory depression	3 (5.00%)	0 (0.00%)	3.068	.080
Wound infection	4 (6.67%)	1 (1.67%)	1.921	.166
Overall complications	19 (31.67%)	6 (10.00%)	8.557	.003

### 3.5. Patient-reported outcomes

Patient satisfaction (9.10 ± 0.80 vs 7.40 ± 1.10) and nursing compliance (92.50 ± 5.20 vs 85.30 ± 6.80) were higher in the intervention group than in the control group (both *P* < .001). The rate of pain-management knowledge acquisition was also higher (93.30% vs 76.70%; *P* = .013) (Table 5).

**Table 5:** Patient-reported outcomes.

Indicator	Control group	Intervention group	*t*/χ^2^	*P* value
Satisfaction score (0–10)	7.40 ± 1.10	9.10 ± 0.80	9.518	<.001
Nursing compliance score (0–100)	85.30 ± 6.80	92.50 ± 5.20	6.787	<.001
Pain-management knowledge rate (%)	76.70%	93.30%	6.172	.013

## 4. Discussion

This study demonstrates that an MMA nursing approach substantially alleviated postoperative pain after hepatectomy, reduced analgesic consumption, accelerated early recovery, lowered complication rates, and improved patient satisfaction and adherence-collectively indicating meaningful clinical value for perioperative care in hepatic surgery.

Inadequate analgesia after hepatectomy can trigger sympathetic activation and amplify the surgical stress response, which in turn impairs cardiopulmonary function and wound healing.^[7]^ In our cohort, VAS scores were consistently lower in the intervention group at 6 to 48 hours, aligning with external evidence. For instance, Kong et al reported in a multi-center randomized trial that MMA reduced 24-hour postoperative pain and opioid use,^[8]^ while Liu et al meta-analyzed hepatic surgery data and found ~1.2-point VAS reductions with MMA compared with single-modality regimens.^[9]^ Mechanistically, MMA likely exerts synergistic effects across peripheral and central pain pathways and mitigates opioid-induced central sensitization; adjunct non-pharmacologic components (e.g., music therapy and relaxation training) further modulate pain perception and enhance satisfaction. Our findings of lower early VAS scores are consistent with this multimodal rationale.

Beyond analgesia, the intervention group exhibited shorter times to 1st ambulation and bowel recovery and a reduced length of stay, which coheres with ERAS principles that adequate pain control facilitates mobilization and pulmonary function. Zhang et al similarly observed a mean 1.3-day reduction in hospitalization with MMA-based protocols after hepatectomy.^[10]^ Importantly, our overall complication incidence decreased from 31.67% in the control arm to 10.00% with MMA nursing, driven primarily by fewer nausea/vomiting events and wound infections, with no cases of respiratory depression in the intervention group-consistent with real-world data showing that opioid-sparing multimodal strategies reduce opioid-related adverse events and overall complications.^[11]^

A distinctive feature of the present work is the emphasis on a collaborative, nurse-centered MMA nursing pathway rather than an anesthesia-only paradigm. Nursing professionals played pivotal roles in scheduled pain assessments, targeted psychological support, and structured early-rehabilitation guidance. Prior literature suggests nurse-led pain programs improve pain recognition and adherence,^[12]^ while continuous assessment plus educational interventions strengthen patient self-management, curb excess medication use, and enhance recovery experience.^[13]^ Our integrated pathway underscores that a standardized, systematized nursing algorithm is integral to the success of MMA strategies.

Unlike studies that focus exclusively on drugs or anesthetic techniques, we evaluated an MMA nursing pathway within the ERAS framework and quantified outcomes spanning pain, functional recovery, complications, and patient-reported endpoints. This “pharmacologic analgesia–nursing intervention–patient engagement” closed-loop model supports clinical feasibility and dissemination and offers additional evidence to refine perioperative nursing pathways for hepatic surgery.

This study has limitations. It was single-center and retrospective with a modest sample size, and we did not stratify by surgical approach (open vs laparoscopic). Additionally, only short-term outcomes were assessed. Future multi-center prospective randomized trials are warranted, ideally incorporating biological markers such as inflammatory mediators and neurotransmitters to elucidate mechanisms by which MMA nursing enhances postoperative recovery.

## 5. Conclusion

MMA nursing-integrating pharmacologic and non-pharmacologic measures with dynamic pain assessment and early rehabilitation-effectively reduces pain and analgesic use, expedites functional recovery, and decreases postoperative complications after hepatectomy, thereby improving patient satisfaction and adherence. This strategy aligns with ERAS concepts, shows favorable safety, and is suitable for wider clinical implementation to optimize perioperative pain management in hepatic surgery.

## Author contributions

**Conceptualization:** Qi Jiang, Ye’ang Qin.

**Data curation:** Qi Jiang, Ye’ang Qin.

**Formal analysis:** Qi Jiang, Ye’ang Qin.

**Funding acquisition:** Qi Jiang, Ye’ang Qin.

**Investigation:** Qi Jiang, Ye’ang Qin.

**Writing – original draft:** Qi Jiang, Ye’ang Qin.

**Writing – review & editing:** Qi Jiang, Ye’ang Qin.
